# Translocator Protein Modulation by 4′-Chlorodiazepam and NO Synthase Inhibition Affect Cardiac Oxidative Stress, Cardiometabolic and Inflammatory Markers in Isoprenaline-Induced Rat Myocardial Infarction

**DOI:** 10.3390/ijms22062867

**Published:** 2021-03-11

**Authors:** Ana Ilic, Dusan Todorovic, Slavica Mutavdzin, Novica Boricic, Biljana Bozic Nedeljkovic, Sanja Stankovic, Tatjana Simic, Predrag Stevanovic, Vera Celic, Dragan Djuric

**Affiliations:** 1 Department of Cardiology, University Clinical Hospital Center “Dr. Dragisa Misovic—Dedinje”, 11000 Belgrade, Serbia; ana.stevanovic15@yahoo.com (A.I.); veracelic@yahoo.com (V.C.); 2 Institute of Medical Physiology “Richard Burian”, Faculty of Medicine, University of Belgrade, 11000 Belgrade, Serbia; t.dusan@hotmail.com (D.T.); slavica.mutavdzin@gmail.com (S.M.); 3 Institute of Pathology, Faculty of Medicine, University of Belgrade, 11000 Belgrade, Serbia; boricic.novica@gmail.com; 4 Institute for Physiology and Biochemistry “Ivan Djaja”, Faculty of Biology, University of Belgrade, 11000 Belgrade, Serbia; najbiljana@yahoo.com; 5 Center for Medical Biochemistry, Clinical Center of Serbia, 11000 Belgrade, Serbia; sanjast2013@gmail.com; 6 Institute of Medical and Clinical Biochemistry, Faculty of Medicine, University of Belgrade, 11000 Belgrade, Serbia; tatjana.simic@med.bg.ac.rs; 7 Department of Anesthesiology, Reanimatology and Intensive Care Medicine, University Clinical Hospital Center “Dr. Dragisa Misovic—Dedinje”, 11000 Belgrade, Serbia; baticaster@gmail.com

**Keywords:** translocator protein, 4′-CIDzp, NO synthase inhibition, myocardial infarction, rat

## Abstract

The possible cardioprotective effects of translocator protein (TSPO) modulation with its ligand 4′-Chlorodiazepam (4′-ClDzp) in isoprenaline (ISO)-induced rat myocardial infarction (MI) were evaluated, alone or in the presence of L-NAME. Wistar albino male rats (b.w. 200–250 g, age 6–8 weeks) were divided into 4 groups (10 per group, total number N = 40), and certain substances were applied: 1. ISO 85 mg/kg b.w. (twice), 2. ISO 85 mg/kg b.w. (twice) + L-NAME 50 mg/kg b.w., 3. ISO 85 mg/kg b.w. (twice) + 4′-ClDzp 0.5 mg/kg b.w., 4. ISO 85 mg/kg b.w. (twice) + 4′-ClDzp 0.5 mg/kg b.w. + L-NAME 50 mg/kg b.w. Blood and cardiac tissue were sampled for myocardial injury and other biochemical markers, cardiac oxidative stress, and for histopathological evaluation. The reduction of serum levels of high-sensitive cardiac troponin T hs cTnT and tumor necrosis factor alpha (TNF-α), then significantly decreased levels of serum homocysteine Hcy, urea, and creatinine, and decreased levels of myocardial injury enzymes activities superoxide dismutase (SOD) and glutathione peroxidase (GPx) as well as lower grades of cardiac ischemic changes were demonstrated in ISO-induced MI treated with 4′-ClDzp. It has been detected that co-treatment with 4′-ClDzp + L-NAME changed the number of registered parameters in comparison to 4′-ClDzp group, indicating that NO (nitric oxide) should be important in the effects of 4′-ClDzp.

## 1. Introduction

Ischemic heart disease (IHD), particularly acute myocardial infarction (MI), represents one of the leading causes of death in developed and developing countries [1]. Acute myocardial infarction (MI) is an ischemic cardiomyocyte necrosis, caused by a disbalance between an inadequate blood flow through the coronary arteries and an increased metabolic oxygen demand [2].

There are many animal models of MI, and the most common used model is isoprenaline-induced MI [3]. Isoprenaline (ISO, isoproterenol, l-(3,4-dihydroxyphenyl)-isopropylaminoethanol hydrochloride) is a synthetic catecholamine and a β-adrenergic agonist that trigger off oxidative stress in the myocardium, and lead to myocardial injury and infarction like necrosis of the cardiac muscle [4].

Translocator protein (TSPO), a protein of 18 kDa, contains 169 amino-acid and is placed in the mitochondrial outer membrane [5]. The TSPO can form a trimeric complex with the 32 kDa voltage-dependent anion channel (VDAC) and 30 kDa adenine nucleotide carrier (ANC) [6] in the outer mitochondrial membrane [7]. At first, it was known as a “peripheral benzodiazepine receptor” (PBR) [8], later it is discovered that it is expressed both centrally and in peripheral organs, mostly in steroid-synthesizing tissues [9], like gonad, adrenal, and brain cells but it is also shown in the kidney and heart. During cardiac ischemia, due to inadequate oxygen concentration, the combination of metabolic imbalance, the excess of cellular Ca^2+^, and the increase of reactive oxygen species (ROS) production, activates mitochondrial membrane permeabilization and the mitochondrial permeability transition pore (mPTP) opening that can contribute to apoptotic and necrotic cell death [8]. TSPO participates in the regulation of mitochondrial functions such as respiration, the membrane potential or opening of mPTP [5]. mPTP is a multiprotein, and its components are the voltage dependent anion channel (VDAC), creatine kinase, cyclophilin D, and TSPO, in addition to adenine nucleotide translocase (ANT) [10]. The protective role of the mPTP against ischemia–reperfusion injury is its inhibition. TSPO ligands could be cardioprotective and some studies reported their valuable effects in different animal models of MI [8,11].

However, many factors take part in ISO-induced myocardial injury and necrosis, but oxidative stress plays a major role [12]. ROS and gasotransmitters like nitric oxide (NO), hydrogen sulfide (H_2_S), and carbon monoxide (CO) have important roles during oxidative stress. They participate in many important cardiovascular physiological and pathophysiological pathways. NO is endogenously produced from L-arginine by a nitric oxide synthase (NOS), however, L-NAME is a non-specific NOS inhibitor which could decrease the NO level. Numerous investigations demonstrated that its application led to an increase of arterial blood pressure, cardiac dysfunction, and MI. Furthermore, there are lot of data that a relationship may exist between NO and H_2_S in the endothelium; number of studies demonstrated that biological effects of H_2_S are regulated by NO and there is existence of their combined roles in hypertension and atherosclerosis [13].

The aim of this study was to investigate the possible cardioprotective effects of modulation of translocator protein (TSPO, also known as “peripheral benzodiazepine receptor”) with its ligand 4′-Chlorodiazepam (4′-ClDzp) in ISO-induced rat MI, alone or during the inhibition of nitric oxide synthesis by L-NAME.

## 2. Results

### 2.1. Induction of Acute MI

The induction of acute MI in rats was based on the model of subcutaneous administration of isoprenaline at a dose of 85 mg/kg/day diluted in saline on two consecutive days. The experimental model of MI in rats was proven in preliminary set of experiments by significant changes in production of biomarkers of myocardial injury in serum during the experimental period, the presence of electrocardiogram (ECG) signs of MI, and histopathological findings (results are not presented here).

### 2.2. Cardiac Ischemic Biomarkers 

**Figure 1:**
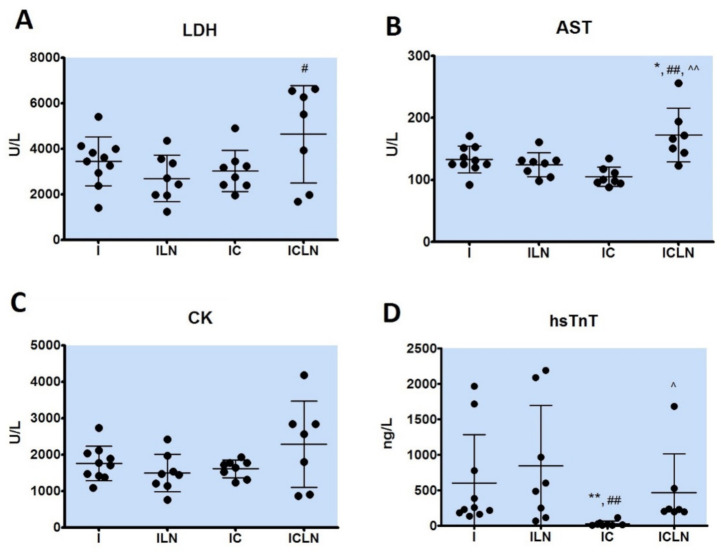
Biochemical parameters of myocardial ischemic injury; serum levels of lactate dehydrogenase (LDH) (**A**), serum levels of aspartate aminotransferase (AST) (**B**), serum levels of creatine kinase (CK) (**C**), serum levels of high-sensitive cardiac troponin T (hsTnT) (**D**). I- ISOPRENALINE (n = 10), ILN-ISOPRENALINE +L-NAME (n = 8), IC- ISOPRENALINE + 4′-CHLORODIAZEPAM (n = 8), ICLN-ISOPRENALINE +4′-CHLORODIAZEPAM + L-NAME (n = 7). ANOVA or Kruskal–Wallis test: *—*p* < 0.05 vs. I, **—*p* < 0.01 vs. I, ^—*p* < 0.05 vs. IC, ^^—*p* < 0.01 vs. IC, #—*p* < 0.05 vs. ILN, ##—*p* < 0.01 vs. ILN.

There was no statistically significant difference among investigated groups in levels of creatine kinase (CK). However, other cardiac injury markers, such as lactate dehydrogenase (LDH), aspartate aminotransferase (AST) and high-sensitive cardiac troponin T (hs cTnT) significantly differ among the groups (Figure 1). Activity of LDH was significantly increased in the ISO + 4′-CIDzp + L-NAME (5516 (1982–6555) U/L) group in comparison to the ISO + L-NAME (2583 (1962–3513) U/L, *p* < 0.05) group. AST values were increased in the ISO + 4′-CIDzp + L-NAME (166 (123.0–256.0) U/L) group in comparison to the ISO (128.7 (123.7–151.9) U/L, *p* < 0.05), ISO + L-NAME (128.1 (98.0–161.1) U/L, *p* < 0.01), and ISO + 4′-CIDzp groups (99.3 (88.1–134.9) U/L, *p* < 0.01). The level of hs cTnT was lower in the ISO + 4′-CIDzp group than in the ISO group (18.5 (9.4–120.0) ng/L vs. 247.9 (136.9–1969.0) ng/L, *p* < 0.01) and ISO + L-NAME (547.4 (150.6–1810.0) ng/L, *p* < 0.01) group, while the levels of hs cTnT were increased in the ISO + 4′-CIDzp + L-NAME group in comparison to the ISO + 4′-CIDzp group (230.0 (199.0–1688.0) ng/L vs. 18.5 (9.4–120.0) ng/L, *p* < 0.05).

### 2.3. Biochemical Parameters in Serum or Plasma

Biochemical parameters in serum or plasma were presented in Table 1. There was statistical difference in the level of Hcy between the groups. A lower serum level of Hcy was measured in the ISO + 4′-CIDZP group in comparison to the ISO group (13.63 (8.47–18.29) μmol/L vs. 26.93 (23.52–29.39) μmol/L, *p* < 0.01). Hcy level was significantly increased in the ISO + 4′-CIDZP + L-NAME in comparison to the ISO + 4′-CIDZP group (18.94 (15.67–31.63) μmol/L vs. 13.63 (8.47–18.29) μmol/L). There were statistically significant differences among the groups in serum levels of uric acid (UA), urea (UREA) and creatinine (CREA). UA was significantly increased in the ISO + 4′-CIDZP + L-NAME group (90.0 (80.0–97.5) μmol/L) than in the ISO, ISO + L-NAME or ISO + 4′-CIDZP groups (66.5 (56.0–72.0) μmol/L, *p* < 0.01, 63.5 (60.2–68.2) μmol/L, *p* < 0.01, 70.5 (65.5–84.5) μmol/L, respectively). The UREA level was significantly lower in the ISO + 4′-CIDZP (7.7 (7.2–9.0) mmol/L) than in the ISO group (10.0 (9.4–10.5) mmol/L, *p* < 0.01), while in the ISO + 4′-CIDZP + L-NAME group (9.8 (8.6–13.6) mmol/L), it was elevated compared to the ISO + 4′-CIDZP group (7.7 (7.2–9.0) mmol/L, *p* < 0.01). CREA was reduced in the ISO + 4′-CIDzp group in comparison to the ISO group (17.0 (15.7–21.0) μmol/L vs. 27.5 (26.7–30.2) μmol/L, *p* < 0.01). Co-application of ISO + 4′-CIDzp + L-NAME significantly increased the CREA level (42.0 (37.5–46.7) μmol/L) in comparison to ISO, ISO + L-NAME, or ISO + 4′-CIDzp (27.5 (26.7–30.2) μmol/L, *p* < 0.01, 24.5 (21.0–27.2) μmol/L *p* < 0.01, and 17.0 (15.7–21.0) μmol/L, *p* < 0.01, respectively).

Total cholesterol (TC) was higher in the ISO + L-NAME group than in the ISO group (1.9 (1.8–2.1) mmol/L vs. 1.7 (1.5–1.9) mmol/L, *p* < 0.05), and even higher in the ISO + 4′-CIDzp + L-NAME group in comparison to all other groups, ISO, ISO + L-NAME, and ISO + 4′-CIDzp (2.2 (2.1–2.6) mmol/L vs. 1.7 (1.5–1.9) mmol/L, *p* < 0.01, 1.9 (1.8–2.1) mmol/L, *p* < 0.05, and 1.6 (1.3–2.1) mmol/L, *p* < 0.05, respectively). High-density lipoprotein cholesterol (HDL-C) was significantly lower in the ISO + 4′-CIDzp + L-NAME in comparison to ISO, ISO + L-NAME, and ISO + 4′-CIDzp + L-NAME groups (0.6 (0.6–0.7) mmol/L vs. 1.0 (0.9–1.1) mmol/L, 1.2 (1.0–1.3) mmol/L, *p* < 0.05, and 0.9 (0.8–1.4) mmol/L, *p* < 0.05, respectively). Triglycerides (TG) were increased in both ISO + 4′-CIDzp and ISO + 4′-CIDzp + L-NAME groups compared to the ISO group (0.48 (0.44–0.48) mmol/L, *p* < 0.01 and 0.47 (0.38–0.80) mmol/L, *p* < 0.05, respectively vs. 0.37 (0.32–0.45) mmol/L).

Alanine aminotransferase (ALT) was increased in the ISO + L-NAME, ISO + 4′-CIDzp, and ISO + 4′-CIDzp + L-NAME group in comparison to the ISO group (61.5 (53.2–81.8) U/L, *p* < 0.01, 53.9 (47.1–60.6) U/L, *p* < 0.01, and 84.0 (75.0–132.0) U/L, *p* < 0.01, respectively vs. 39.1 (36.4–43.1) U/L. ISO + 4′-CIDzp co-treatment increased alkaline phosphatase (ALP) in comparison to the ISO group (209.0 (172.5–242.3) U/L vs. 167.0 (159.5–178.0) U/L, *p* < 0.05), while co-application of ISO + 4′-CIDzp + L-NAME led to even higher values of ALP (258.5 (224.3–279.3) U/L) comparing to the ISO, ISO + L-NAME, and ISO + 4′-CIDzp + L-NAME group (167.0 (159.5–178.0)U/L, *p* < 0.01, 192.5 (125.5–210.8) U/L, *p* < 0.01, and 209.0 (172.5–242.3) U/L, *p* < 0.05, respectively). Alpha-amylase (α-AMY) was higher in both ISO + L-NAME (3094 (2343–3476) U/L, *p* < 0.05) and ISO + 4′-CIDzp groups (3677 (2082–4898) U/L, *p* < 0.05) than in the ISO group (1986 (1773–2546) U/L), while co-application of ISO + 4′-CIDzp + L-NAME led to lower values (1077 (762–1493) U/L, *p* < 0.01). Total protein (TP) concentration was lower in the ISO + 4′-CIDzp + L-NAME group (46.0 (45.0–48.0) g/L) than in the ISO (48.1 (46.7–50.5) g/L, *p* < 0.05) and ISO + L-NAME group (49.0 (47.4–51.2) g/L, *p* < 0.05). Albumin (ALB) was decreased in the ISO + 4′-CIDzp + L-NAME group compared to the ISO, ISO + L-NAME, and ISO + 4′-CIDzp groups (22.0 (22.0–23.0) g/L vs. 31.7 (30.7–32.4) g/L, *p* < 0.01, 32.9 (31.0–35.2) g/L, *p* < 0.01, and 32.7 (29.9–34.2) g/L, *p* < 0.01, respectively). Hemostatic parameters in plasma differ significantly among the groups. Fibrinogen (FIB) was decreased in the ISO + 4′-CIDzp + L-NAME in comparison to the ISO and ISO + 4′-CIDzp groups (2.3 (2.2–2.5) g/L vs. 3.1 (2.6–3.5) g/L, *p* < 0.01, and 3.1 (2.8–3.5) g/L, *p* < 0.01, respectively). Von Willebrand factor (vWF) was increased in the ISO + 4′-CIDzp + L-NAME in comparison to the ISO, ISO + L-NAME, and ISO + 4′-CIDzp groups (247.1 (238.1–310.7)% vs. 212.5 (114.9–228.6)%, *p* < 0.01, 217.3 (205.1–242.8)%, *p* < 0.05, and 201.8 (190.0–219.3)%, *p* < 0.01, respectively).

### 2.4. Cytokine Levels in Serum

**Figure 2:**
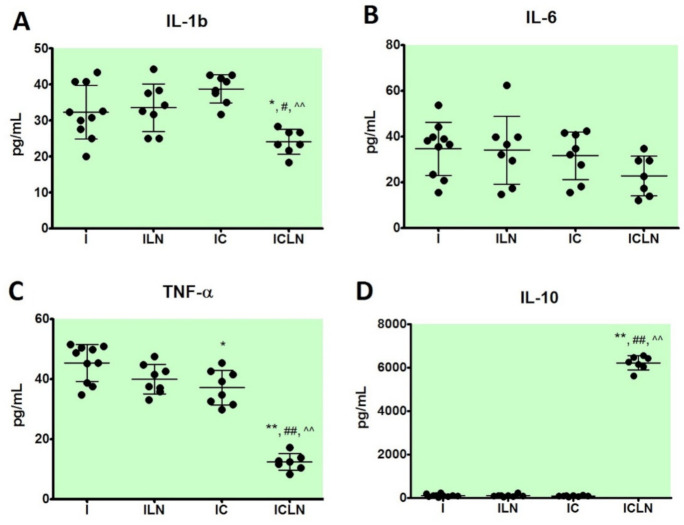
Cytokine levels in serum of rats; serum levels of inteleukin 1b (IL-1b) (**A**), serum levels of interleukin 6 (IL-6) (**B**), serum levels of tumor necrosis factor alpha (TNF-α) (**C**), serum levels of interleukin 10 (IL-10) (**D**). I—ISOPRENALINE (n = 10), ILN—ISOPRENALINE +L-NAME (n = 8), IC—ISOPRENALINE + 4′-CHLORODIAZEPAM (n = 8), ICLN—ISOPRENALINE +4′-CHLORODIAZEPAM + L-NAME (n = 7). Anova or Kruskal–Wallis test: *—*p* < 0.05 vs. I, **—*p* < 0.01 vs. I, #—*p* < 0.05 vs. ILN, ##—*p* < 0.01 vs. ILN, ^^—*p* < 0.01 vs. IC.

Cytokine levels were significantly different between the groups. In the ISO + 4′-CIDzp + L-NAME group, pro-inflammatory cytokines were decreased (IL-1β, IL-6, TNF-α), while anti-inflammatory cytokine was increased (IL-10) (Figure 2). The level of IL-1β was statistically significantly decreased in the ISO + 4′-CIDzp + L-NAME group (23.3 (18.3–28.3) pg/mL) in comparison to all other groups, ISO, ISO + L-NAME, and ISO + 4′-CIDzp (31.6 (25.0–43.3) pg/mL, *p* < 0.05, 33.4 (25.0–44.2) pg/mL, *p* <0.05, and 39.6 (31.7–42.5) pg/mL, *p* < 0.01, respectively). Co-application of ISO + 4′ClDzp + L-NAME significantly reduced the TNF-α level (12.2 (8.3–17.2) pg/mL) in comparison to the ISO, ISO + L-NAME, and ISO + 4′-CIDzp groups (47.0 (34.8–50.9) pg/mL, *p* < 0.01, 39.6 (33.1–47.6) pg/mL, *p* < 0.01, and 37.0 (29.8–45.4) pg/mL, *p* < 0.01, respectively). In addition, there were statistically significant differences between TNF-α levels in the ISO + 4′-CIDzp and ISO groups (*p* < 0.05). Anti-inflammatory cytokine IL-10 was significantly increased in the ISO + 4′-CIDzp + L-NAME (6262.0 (5612.0–6567.0) pg/mL) group in comparison to the ISO, ISO + L-NAME, and ISO + 4′-CIDzp groups (101.3 (30.0–240.0) pg/mL, *p* < 0.01, 101.3 (40.0–230.0) pg/mL, *p* < 0.01, and 87.5 (37.5–125.0) pg/mL, *p* < 0.01, respectively).

### 2.5. Oxidative Stress Parameters

**Figure 3:**
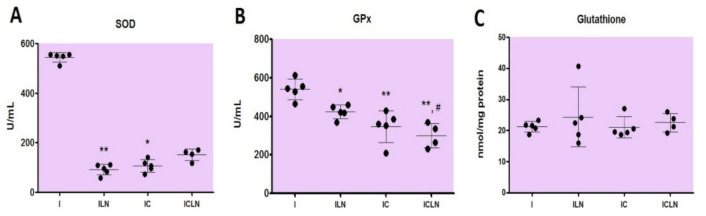
Parameters of oxidative stress in cardiac tissue of rats: superoxide dismutase (SOD) activity (**A**), glutathione peroxidase (GPx) activity (**B**), and total glutathione (GSH) (**C**). I—ISOPRENALINE (n = 5), ILN—ISOPRENALINE +L-NAME (n = 5), IC—ISOPRENALINE + 4′-CHLORODIAZEPAM (n = 4), ICLN—ISOPRENALINE +4′-CHLORODIAZEPAM + L-NAME (n = 4). Anova or Kruskal–Wallis test: *—*p* < 0.05 vs. I, **—*p* < 0.01 vs. I, #—*p* < 0.05 vs. ILN.

In cardiac tissue homogenate, activities of antioxidant enzymes SOD and GPx) were found to be statistically different among the groups (Figure 3A,B), while there were no statistical differences in level of total glutathione (GSH) among the groups (Figure 3C). SOD activities were decreased in the ISO + L-NAME and ISO + 4′-CIDzp groups in comparison to the ISO group (95.5 (59.0–112.4) U/mL, *p* < 0.01, 106.7 (73.0–140.5) U/mL, *p* < 0.05, respectively vs. 550.6 (511.2–556.2) U/mL. GPx activities were decreased in the ISO + L-NAME, ISO + 4′-CIDzp, and ISO + 4′-CIDzp + L-NAME groups in comparison to the ISO group (424.1 ± 34.9 U/mL, *p* < 0.05, 347.6 ± 82.3 U/mL, *p* < 0.01, and 300.6 ± 63.5 U/mL, *p* < 0.01, respectively vs. 541.9 ± 53.3 U/mL). GPx activity was statistically significant lower in the ISO + 4′-CIDzp + L-NAME than in the ISO + L-NAME group (*p* < 0.05).

### 2.6. Histopathological Analysis

There were statistically significant differences in frequency of histopathological findings in comparison between investigated groups. Histopathological grades were described as following: (a) Grade 0—no changes, (b) Grade 1—mild–focal myocyte damage or small multifocal degeneration with a slight degree of inflammatory process, (c) Grade 2—moderate–extensive myofibrillar degeneration and/or diffuse inflammatory process, and (d) Grade 3—severe–necrosis with a diffuse inflammatory process. The histopathological grades of myocardial injuries in the ISO + 4′-CIDzp group had lower ranking compared to the ISO and ISO + 4′-CIDzp + L-NAME groups (Table 2).

## 3. Discussion

As it was mentioned, ISO act on heart via β1- and β2 adrenoceptors, and the result is cardiostimulation, i.e., positive inotropic and chronotropic effects [14]. ISO generates condition of relative ischemia as a result of peripheral vasodilation, tachycardia and coronary hypotension, or hypoxia [15], and cause myocardial ischemia because of cytosolic Ca^2+^ excess [16]. This condition results in oxidative stress with the occurrence of an extreme concentration of free oxygen species [17]. Generally, it could be claimed that specific mechanisms underlying the isoprenaline-induced cardiotoxicity and pathogenesis of acute MI show a clear relationship with processes that are activated like oxidative stress, apoptosis, and inflammation.

TSPO is involved in the regulation of mitochondrial functions such as respiration, the membrane potential or opening of mPTP [5]. One major outcome of mPTP opening, triggered by elevated matrix [Ca^2+^] associated with oxidative stress and depleted adenine nucleotides, is massive swelling of mitochondria, rupture of the outer membrane, and release of intermembrane components that induce apoptosis [18]. Xiaolong et al. has showed that mPTP opening is reduced by 4-ClDzp, and a decreased ROS level could be noticed [19].

In order to establish an appropriate MI experimental model in rats, relationship intended to be established between myocardial injury markers like LDH, AST, CK, and hs cTnT, and cardiac functional parameters on ECG in a preliminary set of experiments. The obtained results confirmed the views of previous studies, that the application of ISO is an adequate approach for experimental induction of MI in rat, and thus represents the “gold standard” for examining the potential cardioprotective effects of pharmacological and non-pharmacological therapeutic modalities, in order to reduce lesions and improve post-infarction myocardial function [20,21].

Myocardial injury markers, such as LDH, AST, and CK are partly sensitive but such as expected, hs cTnT is a highly specific marker for determining the severity of acute MI [22,23]. In the present study, ISO + 4′-CIDzp treatment resulted in significantly lower serum levels of cardiac hs cTnT in comparison to the ISO group, which could also suggest the cardioprotective effects of the 4′-CIDzp (Figure 1). Previous reports suggest that the protective effect of 4′-CIDzp is mediated by its role in inhibition of mPTP opening [24]. Besides, we detected that ISO + 4′-CIDzp treatment induced significantly lower levels of AST and hs cTnT than co-treatment with ISO + 4′-CIDzp + L-NAME (Figure 1). The levels of cardiac markers in the ISO + L-NAME group are slightly lower (LDH, AST, CK) or slightly higher (hs cTnT), but not statistically significant in comparison to the ISO group. There were statistically lower levels of AST in the ISO + L-NAME group in comparison to the ISO + 4′-CIDzp + L-NAME group (Figure 1).

Regarding serum markers of oxidative stress, significantly decreased serum levels of Hcy were detected, which is known as an independent risk factor for MI [25,26], but another marker, uric acid, was not changed. Interestingly, Kannan and Quine [27] showed that increased Hcy levels were found in ISO-induced MI. Hcy can generate endothelial cell injury via oxidant-mediated mechanisms in cellular, animal, and human studies [28,29]. Our results showed that co-treatment with ISO + 4′-CIDzp significantly reduced levels of Hcy in comparison to ISO, possibly due to ROS reduction. A slight increase in serum Hcy level was found in the group co-treated with ISO + 4′-CIDzp + L-NAME, suggesting it was a consequence of decreased NO production. Sugino and Shimada [30] showed that ISO application increased the production of uric acid and decreased renal uric acid excretion. These findings were also observed in an earlier published report [31]. Interestingly, in the present study, a significantly increased level of UA was not found in the group co-treated with ISO + 4′-CIDzp but was found in the group co-treated with ISO + 4′-CIDzp + L-NAME, suggesting again the important role of NO in these effects (Table 1).

In addition, we found significantly decreased serum levels of UREA and CREA in the co-treated ISO + 4′-CIDzp group. Elevated serum levels of UREA and CREA were found in the group co-treated with ISO + 4′-CIDzp + L-NAME, suggesting that decreased NO production led to adverse effects.

Furthermore, lipid status also has a significant role in pathogenesis of MI, by altering the composition, structure, and stability of cellular membranes [32]. In accordance to earlier published reports [33], higher plasma levels of TC and TG were reported in ISO-induced MI. In a previous published report, it was shown that TSPO transported cholesterol from the mitochondrial outer membrane to the mitochondrial inner membrane and promoted mitochondrial cholesterol movement [34]. However, our results demonstrated significantly higher levels of TC in the co-treated ISO + L-NAME group but also in the co-treated ISO + 4′-CIDzp + L-NAME group. Co-treatment with ISO + 4′-CIDzp did not affect serum TC and HDL-C levels but showed significantly higher serum levels of TG.

It was shown that ISO application in the rat experimental model or following intensive stimulation of the sympathoadrenal pathway, the activities of serum enzymes ALT, ALP, and α-AMY were increased, indicating disfunctions of certain organs. This is in accordance with previous published reports [35,36]. We demonstrated significantly increased levels of ALT and ALP and decreased level of α-AMY in the group co-treated with ISO + 4′-CIDzp + L-NAME. In addition, significantly increased serum levels of ALT, ALP, and α-AMY were found in the group co-treated with ISO + 4′-CIDzp.

The findings of a decrease of the serum TP and ALB level in the group co-treated with ISO + 4′-CIDzp + L-NAME is in comparison to the group treated with ISO and the group co-treated with ISO + L-NAME. This is in accordance with a previous published report [31]. A decrease in the level of serum TP in ISO-treated rats could be due to increased free radical and lipid production by ISO.

Von Willebrand factor (vWF) is synthesized in endothelial cells, and increased plasma levels of vWF are in correlation with thrombosis risk and reversely with bleeding risk [37]. In the present study, the plasma level of vWF was statistically higher in the co-treated group with ISO + 4′-CIDzp + L-NAME, which could point to a higher risk of thrombosis (Table 1). Significant increase in plasma levels of fibrinogen is related to an enhanced risk of MI [38,39]. The increased level of fibrinogen in ISO-induced MI rats has been found by Ghazouani et al. [40]. Interestingly, in the present study, the level of fibrinogen was significantly decreased in plasma in the ISO + 4′-CIDzp + L-NAME group in comparison to ISO or ISO + 4′-CIDzp (Table 1), which could point to lower risk of thrombosis.

Three isoforms of NOS can produce NO: eNOS, nNOS, and iNOS. The iNOS is detected in immune cells like macrophages and neutrophils as well as in cardiac myocytes and vascular smooth muscle cells, providing an interesting link for inflammatory processes [41,42]. Basal production of NO takes part in regulation of basal vascular tone, blood pressure, leukocyte–endothelial interactions, inhibits platelet aggregation and neutrophil infiltration. L-NAME restrains L-arginine entering cells and inhibits the interference of any isoforms of NOS with L-arginine. Similar findings were obtained in an earlier published report [43]. The effects of L-NAME treatment could be the result of inhibition of NO biosynthesis.

In the present study, we showed that ISO + 4′-CIDzp reduced MI size; in this group, there were 80% of samples with no histopathological changes and 20% with mild changes. In comparison to the ISO group, where there were 80% with severe changes and 20% with moderate changes. There was also a reduction of MI size in the ISO + 4′-CIDzp group vs. ISO + 4′-CIDzp + L-NAME group which also suggests the cardioprotective role of 4′-CIDzp. There was a slight difference but no significant in ISO + L-NAME co-treatment vs. ISO treatment alone or in comparison to ISO + 4′-CIDzp + L-NAME co-treatment (Table 2). The serum level of hs cTnT is in good relation with the severity of histological changes in ISO + 4′-CIDzp group (Figure 1). TSPO is described as an outer mitochondrial membrane protein and its activity is associated with mitochondrial permeability transition pore (mPTP) opening as well as increased oxidative stress in the ischemic heart [11]. It has been clearly shown that 4′-CIDzp has a cardioprotective role due to the mPTP opening inhibition and diminishing oxidative stress as was in a previously published report [22].

In MI, an important role also belongs to pro-inflammatory cytokines, such as IL-1β, IL-6, and TNF-α, by affecting cardiomyocyte contractility, inflammation, cell death, and endothelial function [44]. These cytokines are also called stress-activated cytokines, characteristic for sterile inflammation present in MI [45,46]. Anti-inflammatory cytokines prevent this process or at best repress severity of the cascade [47], such as IL-10. IL-10 as a worthy inhibitory mediator may be included in determination of the post-MI inflammatory reaction [48,49]. In the atherosclerotic plaque, macrophages, by secreting proinflammatory cytokines, contribute to the local inflammatory responses [50,51,52]. High levels of TSPO expression were detected in activated macrophages [53]. The uptake of [11C] PK11195, as a TSPO radioligand, was investigated in the atherosclerotic plaques. It was higher in inflammatory regions than other sections, as was in a previously published report [53]. The high level of TSPO in plaque macrophagesis represents a diagnostic tool in atherosclerosis through noninvasive PET imaging so it can predict the morphology and pathogenesis of pre-rupture atherosclerosis based on TSPO [52,54]. Serum levels of IL-1β, IL-6, TNF-α, and IL-10 were also determined in the present study. In the serum level of TNF-α, there is a statistical difference in ISO + 4′-CIDzp in comparison to the ISO group (Figure 2). Decrease in serum levels of pro-inflammatory cytokines (IL-1β, TNF-α) and increase in serum level of anti-inflammatory cytokine (IL-10) were detected following co-treatment with ISO + 4′-ClDzp + L-NAME in comparison to the ISO group. This could suggest that ISO + 4′-CIDzp + L-NAME co-treatment affects equilibrium among pro- and anti-inflammatory cytokines. Since the mPTP opening is strongly associated with the TSPO modulation, the results presented here would benefit from a measurement of the mPTP opening and underpin the discussed pathophysiology. It remains unclear why an NOS inhibition on the one hand, partially reverses the cardioprotective effects of 4′-ClDzp treatment, but on the other hand, changes the levels of inflammatory cytokines contrarily, so that it can be a pure epiphenomenon.

Oxidative stress plays a major role in myocardial structural damage and in cardiac remodeling. TSPO is involved in the regulation of oxidative stress [55], as well as in the regulation of the mPTP opening [22,56]. It has been reposted that there is a strong relationship between oxidative stress and extracellular matrix remodeling [56].

In acute and chronic isoproterenol-infused rats, cardiac oxidative stress has been detected [57]. ROS have many sources, such as activation of vascular nicotinamide adenine dinucleo-tide phosphate oxidase, xanthine oxidase, higher angiotensin II and aldosterone levels, auto-oxidation of catecholamines, iNOS, and release of pro-inflammatory cytokines [58]. Proteins are one of the first molecules to endure damage by ROS [59]. Proteins, beside phospholipids, are the main constituents of membranes. High levels of ROS results in the end, in necrosis by peroxidation of lipid members [60]. In this study, the effect of 4′-CIDzp might be due to diminution of oxidative stress, in accordance with an earlier published report [61]. SOD and GPx act in a mutually supportive fashion to detoxify superoxide.

With GSH as a cofactor, SOD catalyzes the reaction of dismutation of superoxide anion (O_2_^-^) into O_2_ and H_2_O_2_, while GPx further reduces H_2_O_2_ into water [62]. GSH also has an additional role in the antioxidant defence system as a cofactor of glutathione transferases, and by scavenging remaining free radicals [63]. Higher levels of SOD and GPx were found in the ISO-treated group, due to tissue damage in acute MI, and probably activated adaptive responses. Similar findings are also observed in earlier published reports [64,65,66]. Oxidative stress is caused by an ineffective antioxidant defence system or extreme production of ROS. Interestingly, treatment of experimental animals with 4′-ClDzp or L-NAME in our ISO-induced MI model, resulted in lower activities of antioxidant enzymes SOD and GPx, while treatment with both of these compounds resulted in lower activity of GPx. The question arises whether these effects are consequence of the protective effects of 4′-ClDzp in terms of lower mitochondrial production of superoxide, hydrogen peroxide, and lipid hydroperoxides, followed by lower activities of antioxidant enzymes. Our data on protective effects of 4′-ClDzp are in line with the study of Brown et al. [67], who showed the same ameliorating effect of 4′-ClDzp in diamide-induced arrhythmias and oxidative stress in normoxic isolated perfused guinea pig hearts. Taken together, these results point to the important role of 4′-ClDzp in improving mitochondrial function in myocardial injury and ischemia. Our results on decreased activities of SOD in treatment with L-NAME should be interpreted in the view of the fact that L-NAME in the heart leads to the fall of SOD activity. The observed changes in antioxidant enzyme activities were not accompanied with differences in total GSH content, suggesting that the synthesis of this compound was not altered. However, the limitation of this study is that we could not distinguish the levels of oxidized and reduced GSH.

## 4. Materials and Methods

### 4.1. Animal Ethics Statement

The study was approved by the Ethical Council for the Welfare of Experimental Animals, Ministry of Agriculture, Forestry and Water Management, Veterinary Directorate, Republic of Serbia (number: 323-07-00412/2020-05, date 22 January 2020.). It was in compliance with recommend legislation (EU Directive for the Protection of the Vertebrate Animals used for Experimental and other Scientific Purposes 86/609/EEC) and the principles of ethics.

### 4.2. Experimental Animals

Wistar albino male adult rats were accommodated in pairs in transparent plexiglas cages with a wood-chip floor. (200–250 g of body weight (b.w.), age 6–8 weeks). Food and water were available ad libitum and the ambient conditions (temperature 21 ± 2 °C, humidity 55% ± 5%, 12 h light–dark cycle with the light period beginning at 7:30 a.m.) were constant.

### 4.3. Experimental Protocol

The model of the rat MI based on the application of ISO in the back at a dose of 85 mg/kg b.w. s.c., total volume of 0.2 mL, given twice with 24 h in-between, on 0th, and 1st day [68]. Experimental animals were divided into 4 groups (total number N = 40; 10 per experimental group), however different number of samples (n) were successfully taken for clinical chemistry and oxidative stress analysis, cytokine determination, and pathohistological analysis. The following substances were applied: 1. ISO 85 mg/kg b.w., 2. ISO 85 mg/kg b.w. given twice subcutaneously (s.c.) with 24 h in-between + L-NAME 50 mg/kg b.w. ip., 3. ISO 85 mg/kg b.w. given twice s.c. with 24 h in-between + 4′-ClDzp 0.5 mg/kg b.w. ip., 4. ISO 85 mg/kg b.w. given twice s.c. with 24 h in-between + 4′-ClDzp 0.5 mg/kg b.w. ip. + L-NAME 50 mg/kg b.w. ip. (during treatment 2 died in this group). MI was confirmed by the increased production of ischemic markers (LDH, AST, CK, hs cTnT) provoked by myocardial injury (serum samples were obtained from rat tail vein, on 0th and 2nd day), the presence of pathognomonic indicators for MI or myocardial ischemia in recorded standard leads electrocardiograms (ECG) (i.e., ST segment elevation (>1 mm) or T wave inversion, respectively), on 0th and 2nd day, and confirmed by cardiac histopathology. At the beginning of the experiment (0th day), and on 2nd day, the sedation was induced by acepromazine 2.5 mg/kg b.w. + 0.01 mL ketamine hydrochloride 10% (100 mg/mL) during ECG recordings and procedure of obtaining blood samples from rat tail vein; at the end of the experimental period on the 2nd day, followed ketamine hydrochloride-induced anesthesia (50 mg/kg b.w.), guillotine sacrifice was performed, and blood and cardiac tissue were sampled for the evaluation of other biochemical, oxidative stress, and inflammatory markers.

### 4.4. Biochemical Parameters Determination in Serum or Plasma

Levels of lipid profile parameters (total cholesterol (TC), high-density lipoprotein cholesterol (HDL-C), triglycerides (TG)), urea (UREA), creatinine (CREA), aspartate aminotransferase (AST), alanine aminotransferase (ALT), creatine kinase (CK), lactate dehydrogenase (LDH), α-amylase (α-AMY), alkaline phosphatase (ALP), total proteins (TP), and albumin (ALB), were measured using spectrophotometry commercial kits (Siemens Healthcare Diagnostics Inc., Newark, NJ, USA) on an automatic analyzer (Dimension Xpand, Siemens, Erlangen, Germany). The serum high-sensitive cardiac troponin T (hs cTnT) levels were measured with a highly sensitive assay using the Roche Cobas e601 automated analyzer (Roche Diagnostics, Mannheim, Germany). Fibrinogen concentration was determined by the modified Clauss assay (Siemens Healthineers Headquarters, Erlangen, Germany) and von Willebrand factor (vWF) activity was determined by particle enhanced assay INNOVANCE^®^ VWF Ac.

### 4.5. Cytokine Levels Determination in Serum

Determination of serum cytokine levels (tumor necrosis factor α (TNF-α), interleukin (IL) -1β, IL-6, and IL-10) was done by ELISA assays (R&D Systems, Minneapolis, MN, USA) according to the manufacturer’s instructions. Blood was drawn from the rat tail vein right before animals were euthanized, samples were preserved at −70 °C until assay. Using a referent standard curve, set up by known amounts of set-provided recombinant cytokines, the concentration of cytokines was measured.

### 4.6. Oxidative Stress Measurements and Preparation of the Cardiac Tissue Samples

Serum homocysteine (Hcy) was measured by competitive immunoassay using direct, chemiluminescent technology on an ADVIA Centaur XP system (Siemens Healthcare Diagnostics, Tarrytown, New York, NY, USA). Serum levels of uric acid (UA) were determined spectrophotometrically using commercial kits (Siemens Healthcare Diagnostics Ltd., Frimley, Camberley, UK) on an automatic biochemical analyzer (Dimension Xpand, Siemens). Other oxidative stress parameters were measured in cardiac tissue homogenate. After isolation, hearts were soaked with saline (0.9% NaCl) and dried on filter paper. Then, cardiac tissue was homogenized in 50 mmol/L RIPA buffer, pH 7.4, with protease inhibitor cocktail (Sigma-Aldrich, St. Louis, MO, USA), and it was centrifuged at 14,000 rpm for 30 min. Samples were kept at −80 °C until the time of analysis. Cu, Zn SOD activity in the homogenates was determined according spectrophotometrically [69,70] based on the ability of SOD to inhibit autooxidation of epinephrine at alkaline pH. One unit of SOD activity was defined as the amount of enzyme which inhibits the oxidation of epinephrine by 50%. GPX activity was measured by the coupled assay procedure [71], and one unit of enzyme activity is expressed as nmol NADPH oxidized/min, assuming 6.22 × 10^3^/L/mol/cm to be the molar absorbency of NADPH at 340 nm. Total glutathione (GSH) was determined spectrophotometrically and expressed as nanomoles per milligram of protein [72]. Protein concentration was determined using a bicinchoninic acid protein assay kit (BCA-1) (Sigma-Aldrich).

### 4.7. Histopathological Analysis

Cardiac tissue was used for histopathological analysis. It was appropriately oriented and cut into sections, 3 mm thickness, transversely. Then, it was fixed by immersion procedure in 4% neutral buffered formaldehyde for 24 h. Afterwards, it was dehydrated with increasing concentrations of alcohol, enlightened in xylene xylol, and molded in paraplast with appropriate device (Tissue Tech II Tissue Embedding Center). Afterwards, every section was cut into slices using appropriate microtome (Leica Reinhart Austria and Leica SM 2000 R). Slices of 5 µm thickness were cut, until the complete cardiac wall thickness become visible. Cardiac slices were stained with proper technique: haematoxylin–eosin (H.E.) and the phosphotungstic acid hematoxylin (PTAH) method. An Olympus BX41 microscope (Tokyo, Japan) with the Olympus C5060-ADU “wide zoom” digital camera was used for all slides. Assessment was made by the image analyzer (ImageJ Q.42 software package). The area of myocardial necrosis was noted for each case. The treatment was unknown to the pathologist. All findings were categorized in the following degrees, to form a histological ranking of myocardial injury: (0) No changes; (1) Mild–focal myocyte damage or small multifocal degeneration with a slight degree of inflammatory process; (2) Moderate–extensive myofibrillar degeneration and/or diffuse inflammatory process; (3) Severe–necrosis with a diffuse inflammatory process [68,73].

### 4.8. Drugs

The following drugs were purchased from Sigma-Aldrich Chemie GmbH, Germany: isoproterenol hydrochloride, 4′-Chlorodiazepam, L-NAME hydrochloride (Nω-Nitro-l-arginine methyl ester hydrochloride). Acepromazine (Neurotranq^R^) was obtained from Alfasan International B.V., JA Woerden, The Netherlands, and ketamine hydrochloride (Ketamidor 10%^R^) from Richter Pharma AG, Wels, Austria.

### 4.9. Statistical Analysis

Values were expressed as mean ± SD or median with interquartile range (IQR). Statistical comparisons between the experimental groups were conducted following tests of normality applied (Shapiro–Wilk), and then one-way analysis of variance (ANOVA), followed by Tukey post hoc test or the Kruskal–Wallis test, Mann–Whitney U test, or Fisher’s exact test depending on the data distribution were used for comparison between the values obtained from experimental groups. SPSS 19.0 for Windows software package was used for statistical analyses. Differences were considered significant at *p* < 0 05.

## 5. Conclusions

In the current study, the putative cardioprotective role of 4′-ClDzp treatment, a negatively regulating ligand of TSPO modulation, was investigated, in the context of ISO-induced myocardial damage in rats, a model of chronic catecholamine activation and consecutive MI. TSPO is described as an outer mitochondrial membrane protein and its activity is associated with mPTP opening as well as increased oxidative stress in the ischemic heart. According to the presented results, the double subcutaneous administration of ISO resulted in acute myocardial injury shown by the increased release of myocardial injury markers, changes in different serum parameters of clinical chemistry, increased parameters for oxidative stress, altered levels of inflammatory cytokines, as well as showing the area of myocardial necrosis. It has been demonstrated that simultaneous 4′-CIDzp treatment led to the reduction in myocardial damage, which was particularly determined by less alteration of certain biochemical markers, i.e., decreased levels of hs cTnT, TNF-α, Hcy, UREA, and CREA, reduced oxidative stress markers SOD and GPx, and improved grading in the histochemical analysis (reduced MI size). This cardioprotective effect could be partially reversed by additional pharmacological NOS inhibition in form of applying the drug L-NAME in parallel. It has been concluded that the cardioprotective effect of 4′-CIDzp after the onset of myocardial ischemia seems, to a certain extent, NO-mediated.

## Figures and Tables

**Table 1:** Biochemical parameters in serum/plasma of rats. Data are expressed as median and IQR. I- ISOPRENALINE (n = 10), ILN- ISOPRENALINE +L-NAME (n = 10), IC- ISOPRENALINE + 4′-CHLORODIAZEPAM (n = 10), ICLN- ISOPRENALINE +4′-CHLORODIAZEPAM + L-NAME (n = 8). Hcy—homocysteine, UA—uric acid, UREA—urea, CREA—creatinine, TC—total cholesterol, HDL C - high-density lipoprotein cholesterol, TG—triglycerides, ALT—alanine aminotransferase, ALP—alkaline phosphatase, α-AMY—alpha-amylase, TP—total protein, ALB—albumin, FIB—fibrinogen, vWF—von Willebrand factor. Mann Whitney U test: *—*p* < 0.05 vs. I, **—*p* < 0.01 vs. I, #—*p* < 0.05 vs. ILN, ##—*p* < 0.01 vs. ILN, ^—*p* < 0.05 vs. IC, ^^—*p* < 0.01 vs. IC.

	I	ILN	IC	ICLN
Hcy (μmol/L)	26.93 (23.52–29.39)	28.6 (22.10–33.61)	13.63 (8.47–18.29) **	18.94 (15.67–31.63) ^
UA (μmol/L)	66.5 (56.0–72.0)	63.5 (60.2–68.2)	70.5 (65.5–84.5)	90.0 (80.0–97.5) **, ##, ^
UREA (mmol/L)	10.0 (9.4–10.5)	9.9 (7.2–9.0)	7.7 (7.2–9.0) **	9.8 (8.6–13.6) ^^
CREA (μmol/L)	27.5 (26.7–30.2)	24.5 (21.0–27.2)	17.0 (15.7–21.0) **	42.0 (37.5–46.7) **, ##, ^^
TC (mmol/L)	1.7 (1.5–1.9)	1.9 (1.8–2.1) *	1.6 (1.3–2.1)	2.2 (2.1–2.6) **, #, ^
HDL-C (mmol/L)	1.0 (0.9–1.1)	1.2 (1.0–1.3)	0.9 (0.8–1.4)	0.6 (0.6–0.7) **, ##, ^^
TG (mmol/L)	0.37 (0.32–0.45)	0.43 (0.37–0.55)	0.48 (0.44–0.48) **	0.47 (0.38–0.80) *
ALT (U/L)	39.1 (36.4–43.1)	61.5 (53.2–81.8) **	53.9 (47.1–60.6) **	84.0 (75.0–132.0) **, #, ^^
ALP (U/L)	167.0 (159.5–178.0)	192.5 (125.5–210.8)	209.0 (172.5–242.3) *	258.5 (224.3–279.3) **, ##, ^
α-AMY (U/L)	1986 (1773–2546)	3094 (2343–3476) *	3677 (2082–4898) *	1077 (762–1493) **, ##, ^^
TP (g/L)	48.1 (46.7–50.5)	49.0 (47.4–51.2)	45.5 (44.3–51.2)	46.0 (45.0–48.0) *, #
ALB (g/L)	31.7 (30.7–32.4)	32.9 (31.0–35.2)	32.7 (29.9–34.2)	22.0 (22.0–23.0) **, ##, ^^
FIB (g/L)	3.1 (2.6–3.5)	2.4 (2.2–3.3)	3.1 (2.8–3.5)	2.3 (2.2–2.5) **, ^^
vWF (%)	212.5 (114.9–228.6)	217.3 (205.1–242.8)	201.8 (190.0–219.3)	247.1 (238.1–310.7) **, #, ^^

**Table 2:** The histopathological grades of myocardial injury in rats. I—ISOPRENALINE, ILN—ISOPRENALINE +L-NAME, IC—ISOPRENALINE + 4′-CHLORODIAZEPAM, ICLN—ISOPRENALINE +4′-CHLORODIAZEPAM + L-NAME. Fisher’s exact test: *—*p* < 0.05 vs. I, ^—*p* < 0.05 vs. IC.

	Grade 0	Grade 1	Grade 2	Grade 3	Σ
I	0 (0%)	0 (0%)	1 (20%)	4 (80%)	5 (100%)
ILN	0 (0%)	0 (0%)	3 (75%)	1 (25%)	4 (100%)
IC *	4 (80%)	1 (20%)	0 (0%)	0 (0%)	5 (100%)
ICLN ^	0 (0%)	0 (0%)	2 (50%)	2 (50%)	4 (100%)
Σ	4 (22.2%)	1 (5.6%)	6 (33.3%)	7 (38.9%)	18 (100%)

Grade 0—no changes; Grade 1—mild–focal myocyte damage or small multifocal degeneration with a slight degree of inflammatory process; Grade 2—moderate–extensive myofibrillar degeneration and/or diffuse inflammatory process; Grade 3—severe–necrosis with a diffuse inflammatory process.

## Data Availability

The data used to support the findings of this study are available from the corresponding author upon request.
